# Timing is everything: structural insights into the disease-linked Kv3 channels controlling fast action-potential firing in the brain

**DOI:** 10.1038/s41467-022-31537-4

**Published:** 2022-07-15

**Authors:** Martin J. Gunthorpe

**Affiliations:** Autifony Therapeutics Ltd, Stevenage Bioscience Catalyst, Gunnels Wood Road, Stevenage, SG1 2FX UK

**Keywords:** Epilepsy, Ion channels in the nervous system, Action potential generation, Receptor pharmacology, Cryoelectron microscopy

## Abstract

Kv3 channels enable neurons to fire at very high frequencies (>100 Hz) which is fundamental to brain development and our ability to make sense of the world at large. Cryo-EM and structure-function studies by Chi et al. now uncover Kv3 channel gating mechanisms and support new precision medicine approaches for CNS diseases.

Humans have a diverse array of more than 70 voltage-gated K^+^ (Kv) channels encoded in their genome, but it is Kv3 channels that have long been known to underlie the ability of some neurons to fire action potentials at very high frequencies ranging from >100 Hz to even 1 kHz for short periods of time (Fig. 1A). Action potentials are a key currency of neuron-to-neuron communication and control the release of neurotransmitters at synapses to fine-tune the activity of target neurons and neuronal circuits in the brain. Such timing and behavior is essential for the proper establishment of the developing nervous system and our ability to make and store memories. Timing is also essential for sensory perception, to bind different sensory stimuli, e.g. sights and sounds, into a single perceptual construct and, consequently, is crucial for our ability to make sense of the world around us. Understanding the control of these processes has long been a goal of neuroscience research. Hence, knowing molecular details about the way in which Kv3 channels help shape the action potential and, through rapid repolarization, prepare neurons to be ready to fire again (and again), is an important step forward in our understanding of our fundamental physiology and the computational power harnessed by our brains. In addition, the growing list of human causal genetic mutations linking Kv3 channel dysfunction to a range of debilitating disorders means that the insights into Kv3 structure and function provided by Chi et al.^1^ will also directly support the development of future precision medicine approaches for the treatment of a wide range of diseases with a high unmet medical need.

**Fig. 1:**
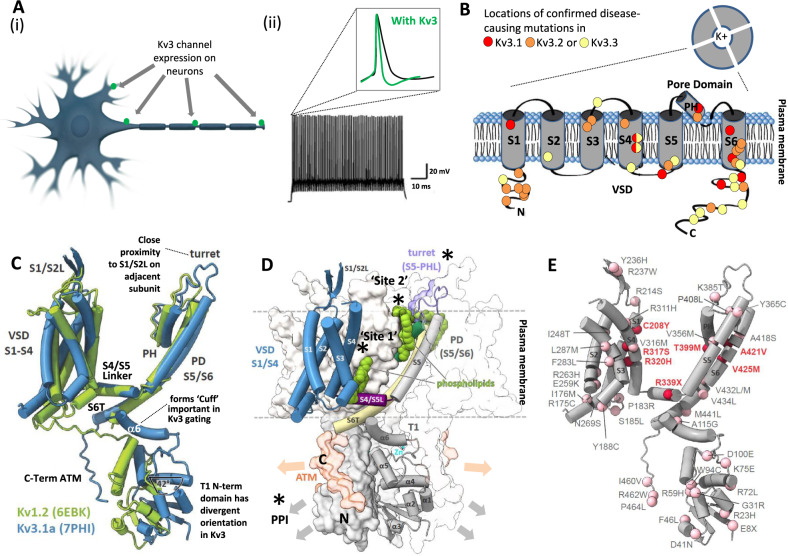
Kv3 channel structure, function and gating. **A** (i) Kv3 channels can be located at the Axon Initial Segment (AIS), along the axon, proximal dendrites and at presynaptic terminals of neurons where they (ii) help shape the ability of the neuron to sustain high frequency trains of action potentials and control neurotransmitter release. Insert shows a single action potential expanded view indicating the impact of Kv3 channels on the action potential waveform driving fast repolarization and keeping refractory periods brief. **B** Schematic of Kv3 channel architecture from above with the central ion conducting pore together with a topology map of a single subunit showing the six transmembrane (TM) domains, N- and C-termini, Voltage-Sensor Domain (VSD), pore helix (PH) and pore lining domain incorporating S5-S6. Four such subunits assemble as tetramers to form a functional channel. Approximate locations of confirmed disease-linked mutations are indicated by colored circles for each Kv3 channel; semicircles indicate coincident mutations in two of the four conserved arginines (R3 and R4) in S4 in Kv3.1 and Kv3.3. **C**–**E** Cryo-EM-determined structural representations of Kv3.1 adapted from Chi et al.^1^ to highlight **C** key differences versus Kv1.2 and channel domains now understood to be important for Kv3 channel behavior as well as **D** locations of the small molecule binding pockets mentioned in the text e.g., turret (purple in **D**) and phospholipid-binding pockets (green in **D**); additional labels refer to Axonal Targeting Motif (ATM), T1 tetramerization domain and S4/S5 Linker. Interacting proteins are likely to bind to the intracellular portion of the channel and form a macromolecular signaling complex in some neurons (yet to be determined) via the C- (orange) and N-terminal (gray) portions of the protein shown in **D**. **E** Summarizes the known human disease-causing mutations in Kv3.1 as per **B** and cited in Table 1 (red) combined with the growing list of likely pathogenic mutations reported in public databases (pink) that map onto the resolved portion of the Kv3.1 structure (all except portions of the T1-S1 linker and aa464 to 511 of the C-terminus).

## Growing numbers of Kv3-linked diseases

Human causal genetic mutations (i.e., DNA alterations encoding amino acid changes in the protein) have now been identified in three out of the four subtypes of Kv3 channel encoded by the *KCNC1-KCNC4* genes (Fig. 1B and Table 1). Consistent with their key physiological role and high expression in the brain and nervous system, the mutations link Kv3 channel dysfunction directly with debilitating CNS diseases including generalized, focal, myoclonic and other epilepsies, as well as intellectual disability, autism, encephalopathies and ataxias. These recent insights have been fueled by increased use of gene sequencing of patients diagnosed with idiopathic epilepsy and other neurodevelopmental disorders, combined with the inclusion of Kv3 genes in diagnostic panels following the first papers indicating their dysfunction linked to disease. For Kv3.1 alone there are now >100 different coding mutations identified in public databases (e.g., ClinVar, GNOMAD etc; Fig. 1E) that are of likely pathogenic relevance; however, only a small fraction of these have so far been characterized in terms of their impact on the channel behavior (e.g. refs. ^2–4^) or in transgenic mouse models (e.g. ref. ^5^). Likely disease-causing mutations have been found extensively throughout the Kv3 channel protein (i.e., in the N- and C-termini as well as VSD and TM domains); so there is clearly much work to do to understand their actual impact on Kv3 function. Since the majority of the reported gene mutations in Kv3.1 are described as being linked to EPM7, it seems likely that the definition of this progressive myoclonic epilepsy disorder will need to broaden beyond that currently described by Muona et al.^6^ for the R320H mutation (aka “MEAK”). In addition, further insights based on the detailed clinical presentation of such patients would clearly be informative. Hence, the full spectrum of disease that may originate from Kv3 channel dysfunction, and that could be addressed therapeutically with a Kv3 modulator, is only just beginning to be appreciated (Table 1).

**Table 1:** Therapeutic potential for Kv3 channel modulators.

Channel	Human genetic disease link (causal mutations identified)	Therapeutic opportunities	References for human disease-linked mutations
Kv3.1	Progressive Myoclonic Epilepsy (PME EPM7), Developmental Encephalopathy without (DE) or with Epilepsy (DEE), Epilepsy of Infancy with Focal Migrating Seizures (EIFMS), autism	PME (EPM7) and related disorders, epilepsy, schizophrenia, Fragile X Syndrome (FXS), autism, hearing disorders, pain	^1–4,15,16^
Kv3.2	Developmental Encephalopathy with Epilepsy (DEE), generalized epilepsy, focal epilepsy, myoclonic-atonic epilepsy, autism	Epilepsy, schizophrenia, hearing disorders, autism, pain	^17–20^
Kv3.3	Spinocerebellar Ataxia (SCA, Type 13), spasticity	Early and late onset spinocerebellar ataxia, hearing disorders	^14,21–23^
Kv3.4	None reported to date	Alzheimer’s Disease and other neurodegenerative diseases, periodic muscle paralysis, pain	

## New insights into Kv3 structure-function

Kv3 ion channels (Kv3.1–Kv3.4) are tetrameric high-voltage activated K^+^ channels that exhibit ultra-fast activation and deactivation kinetics^7–9^. This equips them to be almost silent at rest but exquisitely primed to react to the depolarization associated with action potential generation within micro-seconds to repolarize the neuron so that it can support high levels of repetitive firing (see Fig. 1A, B for additional info). Although there have been some clues as to key domains of the protein responsible for the fast Kv3 channel gating^8^, there are a number of notable new findings based on the cryo-EM Kv3.1a structure at 3.1–3.5 Å combined with mutational analysis and molecular dynamic simulations achieved by Chi et al.^1^.

Like other Kv channels, the Voltage-Sensor Domain (VSD) and Pore Domain/Pore Helix (PD/PH) domains are crucial components of the gating machinery and K^+^ conduction, respectively; although the key S4 helix, which bears a characteristic set of four regularly spaced positive charges in the VSD (conserved arginine residues R1–R4), is in a more inward position in Kv3.1 consistent with a higher threshold for activation. Beyond the VSD, first mechanistic insights into the important role of the cytoplasmic tetramerization “T1” domain in Kv3 channel gating are now evident. Chi et al.^1^ identified a number of unique differences in the orientation of T1 in Kv3.1 compared to other Kvs such as Kv1.2. The entire domain is effectively rotated by >40° in Kv3.1 and the alpha6 helix more so (90°), bringing it in close proximity to elements of the gating machinery (S6T and S4/S5 linker; Fig. 1C, D) and forming a “cuff” impacting open state stability and, through cooperative interactions, providing a means to achieve rapid activation and deactivation. Elegant structure-function studies of key amino acids in this region confirmed the importance of electrostatic interactions between the C-terminal ATM domain and the T1 domain for gating control, a feature also recently shown to be important for the nearby S6T and T1 domains in Kv4.2 channels^10^. An intriguing and unique ability of Kv3 channels to accumulate a reservoir of K^+^ ions in their intracellular vestibule to ensure that repolarizing currents can be sustained was also identified. Also of note was a role of the extended turret region (~9aa extra), which is a unique feature of Kv3 channels, in efficient electromechanical coupling via interactions with the S1/S2 linker and the VSD. Consistent with an important role of all of these interfaces for Kv3 gating noted above, a number of the human disease-linked mutations identified are located in or close to these regions in Kv3.1 and related Kv3 channels.

## Kv3 therapeutics

With respect to the translational potential of this work, it is encouraging that a number of companies are already developing Kv3 modulators and some of these have advanced into clinical development. In addition to the human genetically validated approaches identified, there are also additional opportunities in more prevalent diseases such as schizophrenia, Fragile X syndrome, and Alzheimer’s disease, supported by a growing understanding of the role of Kv3 channels in controlling neuronal activity in relevant cell types and circuits in the brain^9,11,12^. The work by Chi et al. identifies sites on Kv3 channels (asterisks in Fig. 1D) that could provide binding pockets for interaction with small molecule modulators and/or biologics, and these turn out to be near some of the key interfaces identified as critical for Kv3 channel gating. This includes two lipid binding pockets named “Site 1” between S4 and the S4/S5 linker and “Site 2” between the PD and VSD (see Fig. 1D), indicating that Kv3 channel gating, in line with a growing appreciation in the ion channel field, may also be subject to regulation by endogenous lipids. Site 1 was also identified in the preprint by Botte et al.^13^ and, intriguingly, they demonstrated that this pocket could be occupied by a novel small molecule Kv3 channel modulator Lu AG00563. A further binding pocket near the turret and S1/S2 linker domain was also noted by Chi et al. to offer a potential interaction site and its existence may benefit from the extended sequence of this domain already noted in Kv3 channels. The work of Chi et al. was focused on Kv3.1, but an understanding of the structural differences across the Kv3.1–Kv3.4 channel subtypes will surely follow and could even lead to the knowledge-based design of sub-type selective agents that could provide superior therapeutic agents in some circumstances.

There is increasing acknowledgment of the non-canonical role that ion channels play in orchestrating the interaction of other proteins and signaling cascades important for neuronal growth and plasticity. These interactions are influenced by the structural conformation of the channels, as has been shown for Kv3.3 channels which interact with the actin cytoskeleton and Hax-1 to control apoptosis^14^. Thus, greater resolution of the N- and C-termini and the study of macromolecular complexes of the Kv3 channels and their partners will provide new avenues for small molecules and biologics as therapeutic agents by targeting protein epitopes and protein-protein interactions crucial to intracellular signaling (see Fig. 1D).

Validation of specific therapeutic approaches targeting Kv3 channels are already beginning to emerge: e.g., successful attenuation of the symptoms of spinocerebellar ataxia in a mouse model of the Kv3.3-linked human disease (*KCNC3* G592R) using an antisense approach has been demonstrated^5^. Currently there are no approved drugs that specifically target the Kv3 class of ion channel either on the market or in late-stage clinical development. Recent studies highlight direct causal links between Kv3.1, Kv3.2 and Kv3.3 channel dysfunction and disease and it seems likely that it is only a matter of time for Kv3.4. These findings, together with the work of Chi et al., provide increased confidence in the validation and tractability of novel therapeutic approaches for Kv3 channels, including the development of precision medicines. It would therefore seem that the timing is right for recent advances in our understanding of ion channel structure, function and pharmacology to translate into new therapeutic approaches for channelopathies and other diseases with high unmet need.
